# Adult Ewing Sarcoma: Survival and Local Control Outcomes in 102 Patients with Localized Disease

**DOI:** 10.1155/2013/681425

**Published:** 2013-06-11

**Authors:** Safia K. Ahmed, Steven I. Robinson, Scott H. Okuno, Peter S. Rose, Nadia N. Issa Laack

**Affiliations:** ^1^Mayo Medical School, Mayo Clinic, 200 First Street SW, Rochester, MN 55905, USA; ^2^Division of Oncology, Mayo Clinic, 200 First Street SW, Rochester, MN 55905, USA; ^3^Division of Orthopedic Surgery, Mayo Clinic, 200 First Street SW, Rochester, MN 55905, USA; ^4^Division of Radiation Oncology, Mayo Clinic, 200 First Street SW, Rochester, MN 55905, USA

## Abstract

*Objectives*. To assess the clinical features and local control (LC) outcomes in adult patients with localized Ewing Sarcoma (ES). *Methods*. The records of 102 ES patients with localized disease ≥18 years of age seen from 1977 to 2007 were reviewed. Factors relevant to prognosis, survival, and LC were analyzed. *Results.* The 5-year overall survival (OS) and event-free survival (EFS) were 60% and 52%, respectively, for the entire cohort. Treatment era (1977–1992 versus 1993–2007) remained an independent prognostic factor for OS on multivariate analysis, with improved outcomes observed in the 1993–2007 era (*P* = 0.02). The 5-year OS and EFS for the 1993–2007 era were 73% and 60%, respectively. Ifosfamide and etoposide based chemotherapy and surgery were more routinely used in the 1993–2007 era (*P* < 0.01). The 5-year local failure rate (LFR) was 14%, with a 5-year LFR of 18% for surgery, 33% for radiation, and 0% for combined surgery and radiation in the 1993–2007 era (*P* = 0.17). *Conclusion*. Modern survival outcomes for adults with localized ES are similar to multi-institutional results in children. This improvement over time is associated with treatment intensification with chemotherapy and increased use of surgery. Aggressive LC (combined surgery and radiation) may improve outcomes in poor prognosis patients.

## 1. Introduction

The combination of chemotherapy and local control (LC) has significantly improved outcomes for Ewing Sarcoma (ES) [1–5]. Five-year survival rates up to 78% have been achieved in children with localized disease since the addition of ifosfamide and etoposide (IE) chemotherapy [6]. Although historically LC has been problematic, especially in pelvic and axial sites, recent multicenter pediatric trials demonstrate significant improvements in LC with surgery the preferred modality when feasible [6–8]. Modern era 5-year local failure rates (LFRs) are as low as 8%, with 5% for surgery, 25% for RT, and 11% for S + RT [6, 9, 10].

Despite growing literature evaluating the treatment effectiveness in pediatric patients, data assessing outcomes of currently utilized therapy in adults is scarce. Furthermore, conflicting results have been reported with some studies concluding that adult outcomes are worse than pediatric outcomes and others reporting no significant difference [1, 3, 11–15]. As such, the appropriate management of adults remains uncertain. This study was designed to evaluate prognostic factors for survival and LC in 102 adult ES patients ≥18 years with localized disease over a 30-year period. 

## 2. Patients and Methods

### 2.1. Patients

The records of patients ≥18 years with histologically confirmed ES seen at the Mayo Clinic from 1977 to 2007 were reviewed. Patients with metastatic disease at diagnosis, those who presented for recurrent disease, and those with incomplete information on LC were excluded. A total of 102 patients were studied. Pertinent information extracted from patient records included sex, age at diagnosis, site and size of primary tumor, chemotherapy, LC modality, relapse, and survival. Tumor size was based on the greatest reported dimension. 

### 2.2. Definitions and Statistical Methods

Data was analyzed using JMP Statistical Software (Version 8, SAS Institute Inc., Cary, NC). Overall survival (OS) was calculated from the time of diagnosis to date of death or date of last patient contact. Event-free survival (EFS) was calculated from date of diagnosis to first event or date of last patient contact. An event was defined as distant relapse, local relapse, second malignancy, or death, whichever came first. Local failure (LF) was defined as any component of the first relapse at the primary tumor site or at the primary tumor site with distant components. Distant failure (DF) was defined as any distant relapse component of the first relapse. When LF and distant failure (DF) were detected within one month of one another, this was defined as simultaneous failure.

OS, EFS, and failure rate curves were calculated using the Kaplan-Meier method and compared using the log-rank test in univariate analysis. The relative influence of different prognostic factors on survival was estimated with the Cox regression model. Chi-square tests were used to examine associations between categorical variables. A *P* value ≤0.05 was considered significant.

## 3. Results

### 3.1. Followup and Survival

Of the 138 adult ES patients initially identified, 102 patients (74%) presented with localized disease. Patient characteristics are listed in Table 1. Deaths were documented in 44 patients. Median followup for surviving patients was 78 months (range: 4.5 months–27 years). The 5-year OS and EFS were 60% (95% CI, 51–71%) and 52% (95% CI, 43–64%), respectively. 

Univariate analysis was performed to assess the association between pretreatment variables and survival (Table 1). There was no difference in outcomes by sex, age, tumor site, tumor size, and osseous versus nonosseous tumors. Furthermore, there was no association between tumor size and site (*P* = 0.21). 

**Figure 1:**
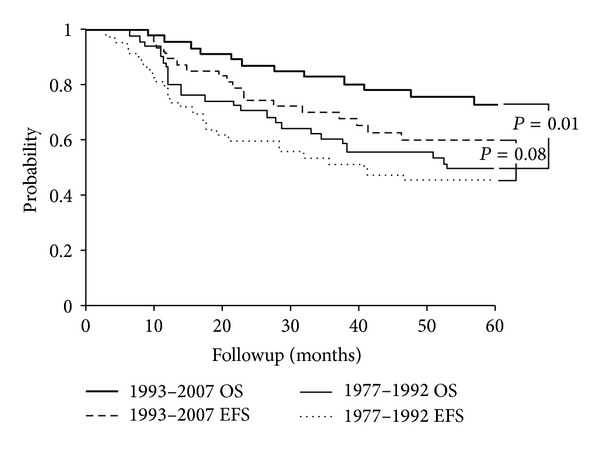
5-year overall (OS) and event-free survival (EFS) by 1977–1992 era and 1993–2007 era (*n* = 102).

As the study spanned a large time period, analysis for two different treatment eras, 1977–1992 (Group A) and 1993–2007 (Group B), was performed. 1992 was chosen as the dividing line for the modern era since this marked a shift in practice towards treating ES per the Intergroup (INT)-0091 trial investigating the inclusion of ifosfamide and etoposide (IE) chemotherapy [18]. The 5-year OS was significantly improved in Group B at 73% versus 49% in Group A (*P* = 0.01) (Table 1) (Figure 1). Though not statistically significant, EFS also trended higher at 60% versus 45% for Group A (*P* = 0.08) (Figure 1). As OS improved for Group B, univariate analysis of prognostic variables restricted to each group was performed. There was no significant difference by variables within the groups (data not shown). 

### 3.2. Chemotherapy

The majority of patients, 95 (94%), received chemotherapy. Chemotherapy regimens are listed in Table 2. Chemotherapy varied according to the time of referral and treatment center policies, as some patients received portions of their treatment at outside institutions. The current chemotherapy standard in ES is dose-intense or interval-compressed alternating vincristine, doxorubicin, cyclophosphamide, ifosfamide, and etoposide (VDC/IE) [6, 16]. In patients with known chemotherapy regimens in our study, 48 patients (54%) received VDC/IE chemotherapy. However, analysis of dose and interval compression could not be done as some patients received portions of their treatment at outside institutions.

**Figure 2:**
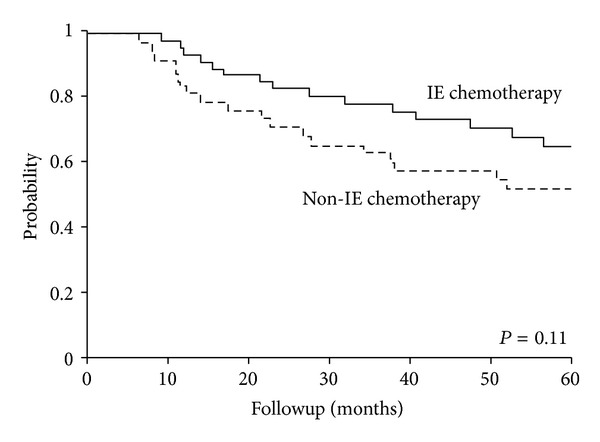
5-year overall survival (OS) by IE chemotherapy versus non-IE chemotherapy (*n* = 89).

Due to these variations, patients were grouped into whether IE chemotherapy was administered or not for analysis. In patients with known chemotherapy regimens, IE chemotherapy was administered to 52 patients (58%). Patients were more likely to receive IE chemotherapy in Group B (88%) than in Group A (12%) (*P* < 0.01). Though not statistically significant, patients who received IE chemotherapy had a slightly higher OS (65% versus 52%, *P* = 0.16) and EFS (55% versus 47%, *P* = 0.41) on univariate analysis (Figure 2). 

### 3.3. Local Control

Surgery was utilized in 43 patients (42%), radiation therapy (RT) in 25 patients (25%), and combined surgery and radiation (S + RT) in 34 patients (33%) (Table 1). For extremity tumors, amputations were performed in six patients: below the knee amputation, 4 patients; toe amputation, 1 patient; and distal phalanx amputation, 1 patient. Rotationplasty was performed in two patients. 

Surgery was more likely to be used in Group B: 58% versus 26% for Group A. Conversely, RT and S + RT were more commonly utilized in Group A (32% and 42%, resp.) than in Group B (17% and 25%, resp.) (*P* = 0.005). Extremity tumors in Group B were more likely to be treated with surgery (70%), pelvic tumors with RT (57%), and axial tumors with surgery (50%) and S + RT (36%) (*P* = 0.03).

Though not statically significant, surgery had the highest survival rates for the entire cohort on univariate analysis (Table 1). The 5-year EFS for surgery was 66% compared to 37% for RT and 48% for S + RT (*P* = 0.15). Analysis for Group B revealed a similar pattern with a 5-year OS rate of 77% for surgery, 63% for RT, and 72% for S + RT (*P* = 0.65). The 5-year EFS rates for Group B were 68% for surgery, 38% for RT, and 58% for S + RT (*P* = 0.30).

### 3.4. Pathological Evaluation of Surgically Treated Patients

**Figure 3:**
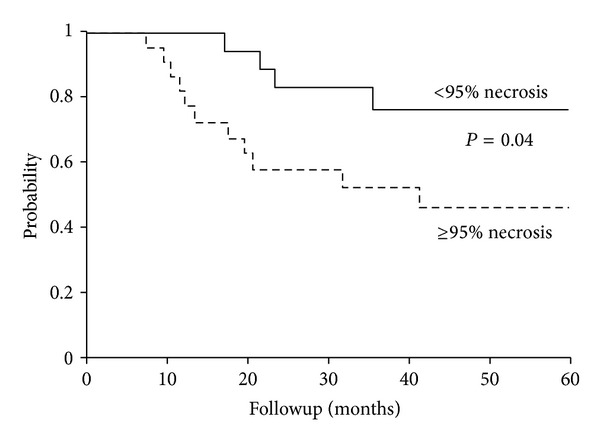
5-year event-free survival (EFS) by histopathologic response to chemotherapy (*n* = 43).

Of the 77 patients who received surgery as a component of LC, 47 patients received neoadjuvant chemotherapy. Excellent responders to chemotherapy were defined as ≥95% necrosis at time of surgery and poor responders as <95% necrosis. Poor histopathologic response to chemotherapy was associated with inferior OS and EFS on univariate analysis (Table 1) (Figure 3). 

Data on surgical margins was available in 68 patients, with clear margins reported in 58 patients (85%) and involved margins reported in 10 patients (15%) as stated in pathology and/or operative reports. Patients with clear surgical margins had significantly higher OS and EFS on univariate analysis compared to patients with involved margins: 69% and 60% versus 25% and 30%, respectively (*P* = 0.008 and 0.009, resp.) (Table 1). There was no significant difference in 5-year LFR by margin status (*P* = 0.77); however, 8/10 patients with involved margins received RT, and no LF was documented in these patients.

### 3.5. RT Dose

RT dose was available in 52 patients. The total dose was <5600 cGy in 42 patients (81%) and ≥5600 cGy in 10 patients (19%). The median dose for definitive RT cases was 5300 cGy (range: 2889–6300 cGy), with a median dose of 4550 cGy (range: 2889–5800 cGy) for Group A and 5580 cGy (range: 5400–6300 cGy) for Group B. 

There was no significant difference in survival outcomes by RT dose in definitive cases on univariate analysis (Table 1). However, though not statistically significant, the 5-year LFR was 0% in patients treated to ≥5600 cGy versus 36% in patients treated to <5600 cGy (*P* = 0.26). Furthermore, though not statically significant, patients treated to ≥5600 cGy had a higher 5-year DFR of 75% compared to a 53% rate observed in patients treated to <5600 cGy (*P* = 0.60). 

### 3.6. Multivariate Analysis

Treatment era was included with variables considered to be prognostic in ES (sex, tumor site, and LC modality) in the Cox regression model for OS and EFS [7, 11, 14, 17]. Tumor size and histopathologic response to chemotherapy were excluded due to incomplete data sets. Treatment era remained an independent prognostic factor for OS: Group A, Hazard Ratio 2.39 (95% CI, 1.14–5.25) (*P* = 0.02) (Table 3).

Since IE chemotherapy was primarily used in Group B, multivariate analysis on the subset of patients with available chemotherapy data was performed (*n* = 89). No association was seen between tumor site, IE chemotherapy, and LC modality and OS or EFS. Treatment era remained an independent prognostic factor for OS: Group A, Hazard Ratio 3.23 (95% CI, 1.15–8.93) (*P* = 0.03). Sex remained an independent prognostic factor for EFS: Male, Hazard Ratio 2.20 (95% CI, 1.04–5.07) (*P* = 0.04).

### 3.7. Patterns of Failure

Relapses were documented in 40 patients with a median time to relapse of 18.2 months (range: 2.9–148 months). The first documented relapse was local failure only (LF) in five patients (12.5%), distant failure (DF) in 28 patients (70%), and LF + DF in seven patients (17.5%). 

The 5-year LFR was 14% (95% CI, 7%–22%). Univariate analysis was performed to assess the association between the variables listed in Table 1 and 5-year LFR and DFR. LC modality emerged as a significant factor for 5-year LFR in the entire cohort. The 5-year LFR for surgery was 17%, 27% for RT, and 0% for S + RT (*P* = 0.04). Though not statistically significant, similar rates were observed in Group B: 18% for surgery, 33% for RT, and 0% for S + RT (*P* = 0.17). 

The 5-year DFR was 37% (95% CI, 26%–46%). No factors were found to be significant on univariate analysis in the entire cohort. Four patients who experienced a DF as the first event experienced an LF afterwards: one year afterwards, 1 patient; two years afterwards, 2 patients; 13 years afterwards, 1 patient. 

### 3.8. Toxicities

One patient experienced extensive ulceration of the lower extremity after irradiation in 1977. This patient refused chemotherapy and received 6400 cGy and below-the-knee-amputation three months later. One patient developed a postradiation histiocytoma 12 years after diagnosis. The patient's ES tumor was in the femur and treated with chemotherapy and RT (5500 cGy).

## 4. Discussion

Only a few studies have reported on adult ES patients, and nearly all of them had small patient populations and/or included patients <18 years of age [11–14]. Our study is the largest reported series to date of localized ES patients exclusively ≥18 years of age. 

Reports in the literature regarding how adults fare in comparison to children are conflicting, with a few studies concluding that prognosis in adults is inferior compared to children [12, 13] and others stating no difference in outcomes between the populations [11, 14]. Moreover, pediatric series include patients up to 30 years of age [6, 18]. Therefore, an exclusive adult series is important as the majority of these patients see adult oncologists.

In our study, adults in Group B showed a significant improvement in survival at 73% compared to 49% in Group A (*P* = 0.01). Though not statistically significant, EFS also trended higher at 60% for Group B versus 45% for Group A (*P* = 0.08). These modern era rates are similar to currently reported 5-year OS and EFS rates of 78% and 70%, respectively, in localized pediatric patients [6]. Furthermore, the improvement in OS remained significant on multivariate analysis even when accounting for increased use of IE chemotherapy (*P* = 0.03). This suggests that the reasons for the improvements in outcomes for adults in the modern era are multifactorial and likely include a combination of factors including adopting pediatric ES chemotherapeutic regimens as well as improvements in local therapy.

Chemotherapy in ES has evolved significantly over time. VAC-based regimens were standard until the first Intergroup Ewing Sarcoma Study (IESS-I) showed superiority of the VACD regimen [3]. Recently, the INT-0091 study showed improved outcomes with the addition of IE [18]. Analysis of chemotherapy regimens in our series revealed a multiplicity of regimens in Group A, including soft tissue protocols versus a more routine use of IE chemotherapy in Group B. However, assorted regimens were still utilized in Group B (Table 2). The variety of regimens employed in our series presumably reflects the uncertainty felt by oncologists in managing adult ES. 

With dose-intensified or interval-compressed VDC/IE chemotherapy the current standard for pediatric ES, the question becomes whether this regimen is also advantageous in adults [6, 16]. Our small numbers, incomplete information on regimens, and inability to assess interval compression made chemotherapy analysis challenging. Therefore, we analyzed chemotherapy with or without IE. Though not statistically significant, there was a clear separation of curves for the groups, with IE chemotherapy having superior outcomes (Figure 2). The subgroup analysis of adults treated on INT-0091 did not support a benefit of IE; however, only 29 adults were studied and were <30 year of ages [18]. The Children's Oncology Group (COG) Protocol AEWS0031 also showed inferior EFS in adults [16]. This study included patients up to 50 years of age, but only 12% of the study population consisted of adults [16]. Nevertheless, we see a higher EFS rate of 56% versus 44% in the INT-0091 study and 47% in the COG AEWS0031 study [18]. These results suggest a positive role for inclusion of IE in adult ES and warrant further study.

LC has also evolved with time, with surgery currently the preferred modality when feasible. In modern pediatric trials, 65-66% of patients receive surgery, 20–23% RT, and 12–15% S + RT [6]. We saw an increased utilization of surgery over time: 26% of patients received surgery in Group A versus 58% in Group B (*P* = 0.005). Though not statistically significant, surgery was associated with the best outcomes, especially in modern years: 77% OS and 67% EFS for Group B (*P* = 0.65 and 0.30, resp.). Pediatric studies have also reported superior outcomes for surgery with 65% OS and 60% EFS [7]. 

These favorable outcomes are thought to be due to a selection bias for smaller and easier to access tumors, such as extremity tumors. Surgery was more likely to be used in the extremities, especially for Group B with 70% of extremity tumors receiving surgery (*P* = 0.03). However, 50% of axial tumors were also treated with surgery in Group B in our series. Furthermore, there was no significant difference in tumor size by site or distribution of tumors compared to children (41–47% extremity, 19–27% pelvis, and 29–35% axial [6, 18]). This suggests that the increasing use of surgery is not only influenced by concerns regarding RT toxicities but by modern surgical advances that have made tumors previously felt to be unresectable, potentially resectable. 

With these improvements, the relative importance of historical prognostic factors may diminish. Location and tumor size were not significant prognostic factors in our study, and newer pediatric data, including the prospective COG study, does not correlate outcomes by location [6]. New prognostic factors are needed to guide clinical decision making. The prognostic value of histopathologic response to chemotherapy is well known, with excellent responses associated with improved survival [11, 14, 19, 20]. Patients with an excellent histopathologic response in our study also correlated with significantly higher OS (84%) and EFS (78%) (Figure 3). This suggests that histopathologic response should be utilized as a prognostic factor for adult ES and guide treatment recommendations. 

We report a 14% 5-year LFR with no significant difference by groups. This is slightly higher than modern reported 8% 5-year LFR in children, likely due to the higher LFR for surgery in our series [6, 9, 10]. The 5-year LFR for surgery in the entire cohort was 17%, with an 18% 5-year LFR in Group B. This rate is higher than pediatric reports of 5% [6, 9, 10]. This again could be a result of small sample size as the surgery LFR for Group B reflects the failure of four patients. Two patients had axial tumors and underwent resection followed by VDC/IE chemotherapy. One patient had involved margins, and one patient had clear margins. Two patients had extremity tumors and received VDC/IE chemotherapy followed by surgery. Both patients had clear margins. One patient had a ≥95% histopathologic response, and one patient had a <95% histopathologic response. 

The RT LFR in our study corresponds to the 25% RT LFR reported in pediatric studies [6, 9, 10]. More importantly, no local failures occurred for patients treated with S + RT (*P* = 0.04), suggesting that S + RT offers an LC advantage over RT or surgery alone in select patients. Patients who received S + RT in our cohort did so because of insufficient necrosis to chemotherapy or marginal/incomplete resection. This is consistent with data from prospective studies which also demonstrate a similar or improved LC rate with S + RT compared to surgery, despite a bias for poorer prognosis patients [8, 21, 22]. Further analysis is needed to identify high risk surgery and RT only patients that may benefit from a combined approach. 

The use of lower RT doses has been associated with a higher LFR; however, dose-response correlation above 4000 cGy is unknown [23]. To investigate whether a dose-response correlation exists, analysis of outcomes for definitive RT cases was performed. Though not statistically significant, no local failures were seen in the four patients treated to ≥5600 cGy; however the 5-year DFR was 75%, whereas the 5-year LFR and DFR were 36% and 53%, respectively, in patients treated to <5600 cGy. Thus, despite a selection bias for poor prognosis patients, there is a suggestion that higher RT doses could improve LC in properly selected patients. Modern imaging, such as FDG PET and diffusion-weighted MRI, may help select patients at higher risk for LF in whom more aggressive local therapy (i.e., higher dose or combined modality) is needed [24]. 

Adults appear to have a low incidence of LC treatment toxicities in our series. The cumulative risk for secondary malignancy development in children is 5–10% over 15–20 years [25, 26]. Only one patient in our study developed a postradiation histiocytoma, and only one patient had a reported RT-induced ulceration. This minimal reported treatment toxicity could be explained by tolerance of a more mature adult skeleton. However, the retrospective nature of this study limits reliability of data. Long-term functional and quality of life data is required to truly assess the effects of LC as our median followup is limited to 78 months.

We recognize that the retrospective nature, small patient population, and incomplete chemotherapy information are limitations of this study. However, it is the first study to detail outcomes in localized adult ES patients ≥18 years of age. Our results suggest that modern era outcomes are similar to children. This appears to be due to intensification of chemotherapy and increasing use of surgery over time. LFR is slightly higher at 14%; however, more aggressive therapy with S + RT may improve outcomes especially in cases with marginal resection and poor histopathologic response. The low occurrence of LC toxicities observed further supports the role of more aggressive local therapy. Our data showing similar modern era outcomes to children supports the inclusion of adults in pediatric trails. In the currently open COG AEWS1031, patients up to age 50 are eligible for enrollment.

## Figures and Tables

**Table 1:** Patient characteristics and univariate analysis for overall  (OS) and event-free survival  (EFS)  (*n* = 102).

Variable	No. of patients (%)	5-year OS (%)	*P*	5-year EFS (%)	*P*
Sex					
Male	69 (68)	58	0.30	47	0.12
Female	33 (32)	67		65	
Age, years					
Median	27.56				
Range	18–60				
18–25	46 (45)	57	0.68	50	0.87
26–35	32 (31)	63		53	
36+	24 (24)	64		56	
Primary tumor site					
Extremities	51 (50)	60	0.90	52	0.95
Pelvis	17 (17)	58		53	
Axial	34 (33)	63		53	
Primary tumor site					
Nonosseous	32 (31)	60	0.65	52	0.72
Osseous	70 (69)	60		52	
Tumor size					
<8 cm	38 (58)	60	0.96	50	0.77
≥8 cm	27 (42)	60		57	
Treatment era					
1977–1992	50 (49)	49	0.01*	45	0.08
1993–2007	52 (51)	73		60	
LC modality					
Surgery	43 (42)	71	0.27	66	0.15
RT	25 (25)	49		37	
S + RT	34 (33)	57		48	
IE chemotherapy					
No	37 (42)	51	0.11	45	0.30
Yes	52 (58)	66		56	
Histopathologic response to chemotherapy					
<95% necrosis	24 (56)	49	0.04*	46	0.04*
≥95% necrosis	19 (44)	83		77	
Surgical margins					
Clear	58 (85)	69	0.008*	60	0.009*
Involved	10 (15)	25		30	
RT dose (cGy) (definitive RT only)					
<5600	19 (83)	50	0.85	38	0.81
≥5600	4 (17)	50		25	

*Statistically significant.

**Table 2:** Chemotherapy regimens (*n* = 102).

Chemotherapy	Group A (*n* = 50)	Group B (*n* = 52)
VDC/IE	5	43
VACD	9	
VDC	8	
VAC	3	
MAP	3	
MAP with RT followed by VDD alternating with VDC	5	
MAP, IE		3
Unknown regimen	4	2
VAC alternating with VDD	1	
VC	2	
VDC, 5FU	1	
VDCD	1	
IE	1	
DI		1
CCT		1
MDCI		1
DDI		1
No chemotherapy administered	6	
No chemotherapy information	1	

VDC/IE: vincristine, doxorubicin, cyclophosphamide, ifosfamide, etoposide; VACD: vincristine, actinomycin D, cyclophosphamide, doxorubicin; VDC: vincristine, doxorubicin, cyclophosphamide; VAC: vincristine, actinomycin D, cyclophosphamide; MAP: mitomycin, Adriamycin, cisplatin; VDD: vincristine, doxorubicin, dacarbazine; VDCD: vincristine, doxorubicin, cyclophosphamide, dacarbazine; DI: doxorubicin, ifosfamide; CCT: cyclophosphamide, carboplatin, thiotepa; MDCI: mitomycin, doxorubicin, cisplatin, ifosfamide; DDI: doxorubicin, dacarbazine, ifosfamide.

**Table 3:** Multivariate analysis for overall (OS) and event-free survival (EFS) (*n* = 102).

Variable	OS		EFS	
	Hazard Ratio (95% CI)	*P*	Hazard Ratio (95% CI)	*P*
Sex				
Female	1	—	1	—
Male	1.58 (0.76–3.55)	0.23	1.71 (0.86–3.66)	0.13
Site of primary tumor				
Extremities	1	—	1	—
Pelvis	1.08 (0.42–2.51)	0.86	0.95 (0.40–2.08)	0.91
Axial	1.19 (0.51–2.68)	0.68	1.12 (0.53–2.32)	0.77
Treatment era				
1993–2007	1		1	
1977–1992	2.39 (1.14–5.25)	0.02*	1.62 (0.84–3.20)	0.15
LC modality				
Surgery	1	—	1	—
RT	1.32 (0.54–3.29)	0.54	1.53 (0.67–3.57)	0.31
S + RT	1.28 (0.56–2.96)	0.56	1.44 (0.68–3.11)	0.34

*Statistically significant.
